# Atypical delta-band phase consistency and atypical preferred phase in children with dyslexia during neural entrainment to rhythmic audio-visual speech

**DOI:** 10.1016/j.nicl.2022.103054

**Published:** 2022-05-20

**Authors:** Mahmoud Keshavarzi, Kanad Mandke, Annabel Macfarlane, Lyla Parvez, Fiona Gabrielczyk, Angela Wilson, Usha Goswami

**Affiliations:** Centre for Neuroscience in Education, Department of Psychology, University of Cambridge, Cambridge CB2 3EB, United Kingdom

**Keywords:** Developmental dyslexia, Neural phase entrainment, Low frequency bands, Rhythmic audio-visual speech

## Abstract

According to the sensory-neural Temporal Sampling theory of developmental dyslexia, neural sampling of auditory information at slow rates (<10 Hz, related to speech rhythm) is atypical in dyslexic individuals, particularly in the delta band (0.5–4 Hz). Here we examine the underlying neural mechanisms related to atypical sampling using a simple repetitive speech paradigm. Fifty-one children (21 control children [15M, 6F] and 30 children with dyslexia [16M, 14F]) aged 9 years with or without developmental dyslexia watched and listened as a ‘talking head’ repeated the syllable “ba” every 500 ms, while EEG was recorded. Occasionally a syllable was “out of time”, with a temporal delay calibrated individually and adaptively for each child so that it was detected around 79.4% of the time by a button press. Phase consistency in the delta (rate of stimulus delivery), theta (speech-related) and alpha (control) bands was evaluated for each child and each group. Significant phase consistency was found for both groups in the delta and theta bands, demonstrating neural entrainment, but not the alpha band. However, the children with dyslexia showed a different preferred phase and significantly reduced phase consistency compared to control children, in the delta band only. Analysis of pre- and post-stimulus angular velocity of group preferred phases revealed that the children in the dyslexic group showed an atypical response in the delta band only. The delta-band pre-stimulus angular velocity (−130 ms to 0 ms) for the dyslexic group appeared to be significantly faster compared to the control group. It is concluded that neural responding to simple beat-based stimuli may provide a unique neural marker of developmental dyslexia. The automatic nature of this neural response may enable new tools for diagnosis, as well as opening new avenues for remediation.

## Highlights

• Children with and without dyslexia showed consistent phase entrainment.
• Dyslexic children had significantly reduced delta band phase consistency.
• Dyslexic children had a different preferred phase in delta compared to controls.
• The dyslexic brain showed faster pre-stimulus delta band angular velocity.

## 1 Introduction

Studies of the neural encoding of speech by children with developmental dyslexia are few in number but are largely consistent in revealing atypical encoding of low-frequency speech information in a range of languages (English: Power et al., 2016, Power et al., 2016, Di Liberto et al., 2018, during sentence and story listening respectively; Spanish: Molinaro et al., 2016, during sentence listening; and French: Destoky et al., 2020, during a speech-in-noise paradigm). Low frequency information in the speech signal <10 Hz is important for perceiving speech rhythm and prosody (Greenberg, 2006). The atypical encoding of low frequency information in speech by children with dyslexia is proposed to underlie the phonological ‘deficit’ that characterises children with dyslexia across languages (Goswami, 2011; ‘Temporal Sampling’ theory). Children with dyslexia fail to acquire efficient reading and spelling skills despite adequate tuition and an absence of overt sensory and/ or neural deficits (Lyon et al., 2003), and numerous behavioural studies reveal associated difficulties in identifying and manipulating phonological units in oral tasks at all linguistic levels: prosody, syllable, onset-rime and phoneme (Snowling, 2000, Goswami, 2015). Phonological difficulties can even be identified in infants and toddlers at family (genetic) risk for dyslexia (Kalashnikova et al., 2019, Kalashnikova et al., 2020). If reading is defined as the cognitive process of understanding a visual code for *spoken* language (Ziegler and Goswami, 2005), then these multiple difficulties with the phonology of the spoken language can be understood developmentally*.* Put simply, individual differences in the efficiency of learning the visual code for speech cannot be separated from the pre-existing efficiency of the child’s spoken language skills (see Goswami, 2022, for extensive review). The visual codes used for different orthographies are not neutral visual stimuli. Rather, they are culturally-specific codes for representing the sound structures (phonology) of words in different languages.

Indeed, while dyslexia has been found in all languages so far studied, its manifestation can differ with orthography. In consistent alphabetic orthographies where learning print can help to specify phonology, for example German, dyslexic children read slowly and laboriously but accurately. In inconsistent alphabetic orthographies where learning print is only a partial guide to phonology, for example English, dyslexic children are both slow and inaccurate readers (Ziegler and Goswami, 2005). Learning print rarely helps children to learn the prosodic level of phonology nor the syllabic segmentation of words, hence it is interesting that speech-based studies of neural encoding in dyslexia find differences focused on the delta and theta neural oscillatory bands (0.5 – 4 Hz and 4 – 8 Hz; Power et al., 2013, Power et al., 2016, Molinaro et al., 2016, Di Liberto et al., 2018, Destoky et al., 2020). In the adult neural speech encoding literature, delta band encoding is most typically related to prosodic, intonational and phrasal features of the speech signal (Ding and Simon, 2014), while theta band encoding helps to identify the onsets of syllables, which may contribute to speech parsing (Ding and Simon, 2014, Di Liberto et al., 2015, Keshavarzi et al., 2020). In terms of learning phonology, impaired neural encoding of lower frequency information in the speech signal would thus be expected to affect the recovery of prosodic and syllabic structure (including the identities of strong versus weak syllables) from the speech signal of any language.

Although these developmental studies of neural speech encoding all reveal impaired encoding of low frequency speech information, identifying which mechanisms of encoding may be atypical has proved difficult. This is important for the design of remedial programmes, yet the mechanisms contributing to the identified asynchrony in speech-brain alignment are seldom investigated further. A related neural literature using the ASSR (amplitude steady state response, with amplitude-modulated [AM] noise as the input) as the dependent measure has proved confusing regarding impaired mechanisms. Studies of both children and adults show ASSR impairments in dyslexia, but at a range of different temporal rates, with some studies in the same languages providing directly contradictory information (Lizarazu et al., 2021; for a recent review). One way forward regarding identifying impaired mechanisms may be to focus on using speech rather than AM noise as the input, and to study the neural encoding of rhythmic and therefore temporally-predictable speech stimuli.

Although spoken languages are thought to differ in their linguistic rhythm types, for example exhibiting stress-timing (where syllables are stressed at equal temporal intervals, regardless of the number of intervening syllables) versus syllable-timing (where syllables have approximately equal durations), linguistic analyses of speech production across many languages show that stressed syllables are produced by human speakers approximately every 500 ms, providing a reliable temporal marker for the prediction of important speech information (Dauer, 1983, Kotz et al., 2009). To gain further information regarding neural mechanisms of speech processing in dyslexia, the cortical tracking ability of the dyslexic brain for temporally-predictable speech information may thus be able to provide novel insights. Indeed, a focus on rhythmic speech has been found useful for studying speech encoding by infants (Gibbon et al., 2021). It is notable that Babytalk or infant-directed speech (IDS) shows exaggerated prosody and very strong rhythms (Fernald et al., 1989, Jusczyk et al., 1993, Leong et al., 2017). EEG studies with typically-developing infants reveal enhanced delta band cortical tracking of IDS compared to theta band tracking (Attaheri et al., 2022). This may suggest that initial language encoding is enhanced by accurate delta band cortical tracking, which would support learning to predict the acoustic landmarks in speech that occur approximately every 500 ms across languages, namely stressed syllables. If accurate delta band cortical tracking were impaired in dyslexia from birth, the entire linguistic system could be expected to develop differently (Goswami, 2019). Difficulties in the automatic extraction of prosodic and syllabic structure would have inevitable consequences for learning about phonemes when print is acquired. Studies with children and with illiterate adults show that awareness of phonemes as linguistic units is a consequence rather than a precursor of learning to read (Ziegler and Goswami, 2005, Goswami, 2015).

In the current study, we employ a rhythmic speech entrainment task first developed by Power et al., 2012, Power et al., 2013. Power et al. (2013) designed a speech paradigm based on rhythmic repetition of the syllable “ba” at a 2 Hz rate (one syllable every 500 ms). English-speaking children with dyslexia either saw a ‘talking head’ repeating “ba”, with both visual and auditory information present (audio-visual or AV condition), saw the talking head without sound, so that only visual information was present (V), or heard the auditory stimulus stream in the absence of visual stimulation (A). The children were asked to detect occasional rhythmic violations in each condition (A, V, AV) in an adaptive paradigm. The degree to which the violator was out of the isochronous rhythm was varied adaptively depending on how well the child was doing in the task using a 3-down 1-up staircase procedure. The adaptive procedure was employed to equate task engagement and task performance across participants, meaning that subsequent neural comparisons between children with dyslexia and control children were not complicated by differential task performance. Here we study younger children with dyslexia, in order to see whether group differences in the speech listening task would also be present at the onset of reading (studying 9-year-olds instead of the 13-year-olds studied by Power and colleagues). The 9-year-olds with dyslexia in the current report had very undeveloped reading skills, reading on average at the level of a typically-developing 7-year-old (the children had an average reading age of 85.5 months, which was over 2 years behind their average age of 110.7 months, see Table 1). We also include a larger group of participants (30 children with dyslexia instead of 11 children), and employ new methods of data analysis (pre-stimulus angular velocity, event-related potentials [ERPs]). The novel analyses are aimed at identifying which mechanisms of neural speech encoding may be atypical in dyslexia and how this may relate to reduced prediction of critical acoustic landmarks in speech such as the placement of stressed syllables.

**Table 1:** Group characteristics expressed as mean and (S.D.) for children with dyslexia and age-matched control children, also showing group characteristics for the N = 21 children with dyslexia who scored lowest on the phonological and behavioural measures.

	Dyslexic	Age-Matched Control	N = 21 Dyslexic
N	30	21	21
Age (months)	110.7 (5.6)	109.3 (5.4)	110.7 (6.1)
WISC Similarities	9.9 (2.1)*	11.1 (1.7)	9.5 (2.2)*
WISC Matrix reasoning	9.6 (2.8)	9.1 (3.0)	9.1 (2.8)
BPVS SS	103.6 (11.5)	103.3 (11.0)	104.0 (11.8)
BAS Reading SS	81.0 (8.0) ***	99.5 (6.2)	78.1 (6.7)***
BAS Reading Age in months	85.5 (11.0) ***	106.5 (11.7)	81.8 (8.5) ***
BAS Spelling SS	79.9 (7.5) ***	97.1 (6.1)	77.4 (5.9) ***
TOWRE SWE SS	79.5 (12.8) ***	101.1 (7.7)	75.4 (12.4) ***
TOWRE PDE SS	79.2 (10.9) ***	98.0 (8.6)	75.0 (9.6) ***
PhAB Rhyme SS	92.6 (11.7) ***	102.4 (5.9)	90.4 (11.8) ***
PhAB Phoneme SS	97.6 (9.8) **	105.1 (9.4)	93.4 (6.7) ***
PhAB RAN objects SS	91.9 (15.0) *	101.2 (13.0)	86.6 (12.9) ***
PhAB RAN digits SS	85.8 (15.8) **	97.4 (13.3)	79.0 (10.5) ***
Rise time sine ms	174.7 (81.7)	139.7 (84.5)	191.2 (82.2)+
Rise time SSN ms	221.9 (56.7)	215.0 (54.9)	237.7 (50.4)
Rise time “ba” ms	101.5 (43.8)*	70.8 (34.6)	96.2 (43.4)*

Note. ***p < 0.001; **p < 0.01; *p < 0.05; +p = 0.052. WISC FSIQ = Wechsler Intelligence Scale for Children, Scaled Scores, scaled mean = 10, SD = 3; BPVS = British Picture Vocabulary Scales, SS = standardised mean score = 100; BAS = British Ability Scales, SS = standardized score = 100; TOWRE SWE = Test of Word Reading Efficiency Sight Word Efficiency Scale, SS = standardized score = 100; TOWRE PDE = Phonic Decoding Efficiency Scale, SS = standardized score = 100; PhAB = Phonological Awareness Battery, SS = standardised mean score = 100; RAN = Rapid Automatized Naming; Rise time = threshold in rise time task in ms; SSN = speech-shaped noise.

Power et al. (2013) reported that both children with dyslexia and age-matched controls showed significant neural entrainment in both the delta and theta bands, however strength of entrainment and response power were not statistically different between groups. Nevertheless, as a group the children with dyslexia showed a significantly different preferred phase in terms of inter-subject coherence in the delta band for the AV and A conditions compared to age-matched controls. No group differences were found regarding the theta band. EEG phase patterns reflect the selectivity of neural responding, with populations of neurons more likely to fire at specific phases in response to an auditory stimulus (Ng et al., 2013). Neural oscillations also entrain to stimuli at differing preferred phases depending on whether they are being attended to or being ignored (Lakatos et al., 2008, Besle et al., 2011, Horton et al., 2013, Keshavarzi et al., 2021). Power et al.’s data suggest that there is an optimal or preferred phase of entrainment which is necessary for accurate and efficient processing of rhythmic speech, and that this preferred phase is different in the (child) dyslexic brain when speech is the input. This could be expected to reduce the efficiency of linguistic processing and would presumably impede the automatic extraction of phonology.

A non-speech rhythmic entrainment study with dyslexic adults extended these findings (Soltész et al., 2013). Soltész et al.’s experimental paradigm also required participants to respond to an oddball, this time a white noise tone in a stream of sine tones presented isochronously at 2 Hz. A condition with a 1.5 Hz tone stream was also employed. Soltész et al. found that neural entrainment (inter-trial coherence, or phase locking) was significantly reduced in the dyslexic participants in both conditions, even though they were as fast and as accurate as the control adults in the button-press paradigm. Further analyses of the instantaneous phase of the delta oscillation just prior to stimulus delivery showed that the phase of entrainment was significantly related to reaction time in the rhythmic paradigm, but for the control participants only. Whereas the control participants showed faster responses in the rising phase of the oscillation, as would be expected if the phase of entrainment contributes to accurate and efficient stimulus processing (Stefanics et al., 2010), the dyslexic participants showed no relationship between oscillatory phase and behaviour. These data suggest that the adults with dyslexia were achieving matched performance to the controls in the rhythmic responding paradigm by using other cognitive or sensory processes. A further analysis by Soltész and her colleagues of associated ERPs showed significantly less neural preparation for the upcoming and totally predictable next tone stimulus in the adults with dyslexia (Soltész et al., 2013). Accordingly, temporal prediction mechanisms regarding rhythmic events were impaired in the (adult) dyslexic brain.

In the current study, our aim was to build on the insights provided by Power et al. (2013) and by Soltész et al. (2013) by conducting a new study of children with developmental dyslexia and significantly extending the analysis methods used previously. By using speech rather than tones as the input, we can study whether the insights regarding neural preparation and instantaneous phase reported by Soltész et al. (2013) extend to children and to speech. We also add novel analyses of pre-stimulus angular velocity, which was not studied by Power et al. (2013) nor by Soltész and her colleagues. As our children were 4 years younger than those participating in the study by Power et al. (2013), we used the AV condition from that study only, to reduce the length of the experiment. A priori, we expected to replicate Power et al.’s findings of significant entrainment for all children, accompanied by the dyslexic brain showing a different preferred phase to 2 Hz rhythmic speech. Regarding our novel measures for children of angular velocity and pre-stimulus neural preparation, we predicted impairments in dyslexia in the delta band only, not in the theta band. This would be consistent with prior speech encoding studies in English and Spanish based on sentences (Power et al., 2016, Molinaro et al., 2016), in which atypical processing was strongest in the delta band.

## 2 Material and methods

### 2.1 Participants

Twenty-one typically developing children (mean age of 109.3 ± 5.4 months) and thirty children with developmental dyslexia (mean age of 110.7 ± 5.6 months) who were participating in an ongoing longitudinal study of 120 children with and without developmental dyslexia (2018–2023) volunteered for the EEG study. The children were assigned as dyslexic or typically-developing on the basis of standardized reading and spelling measures taken in 2018. Children were assessed experimentally using the British Ability Scales standardized tests of reading and spelling (Elliott et al., 1996), and the Test of Word Reading Efficiency word and nonword scales (TOWRE, Torgesen et al., 1999), and were included in the study if they scored at least 10 standard points below the test norm of 100 on at least two of these four measures. The EEG data reported here was collected between September 2019 and February 2020, at which point Covid-19 necessitated the cessation of testing part-way through the study. This meant that the planned matched group design (30 children with dyslexia, 30 age-matched controls, 30 reading-level matched controls) could not be completed, and there were unequal group sizes. Children with dyslexia were recruited via learning support teachers, and only children who had no additional learning difficulties (e.g., dyspraxia, ADHD, autistic spectrum disorder, developmental language disorder [DLD]), a nonverbal IQ above 84, and English as the first language spoken at home were included. The absence of additional learning difficulties was based on school and parental reports and our own testing impressions. Participants were attending state schools (equivalent to US public schools) situated in a range of towns and villages near a university town in the United Kingdom. Most families were Caucasian and of lower class or middle-class regarding income. All children received a short hearing screen using an audiometer. Sounds were presented in both the left or right ear at a range of frequencies (250, 500, 1000, 2000, 4000, 8000 Hz), and all children were sensitive to sounds within the 20 dB HL range. All participants and their parents gave informed consent for the EEG study in accordance with the Declaration of Helsinki, and the study was approved by the Psychology Research Ethics Committee of the University of Cambridge.

### 2.2 Background measures: standardised tests of reading, spelling, phonology, vocabulary, rise time sensitivity and IQ

Standardised tests of written and spoken language were used in schools to assess cognitive development prior to the imaging session and are summarised in Table 1. Reading and spelling were assessed using the British Ability Scales (BAS, an untimed test, Elliot et al., 1996) and the Test of Word Reading Efficiency (TOWRE, a timed test, Torgesen et al., 1999). Four subscales from the Wechsler Intelligence Scale for Children (WISC-V, Weschler, 2016) were administered during the first years of the study. These included two verbal (vocabulary and similarities) and two non-verbal (block design and matrix reasoning) scales. At the time of EEG data collection, the WISC similarities and WISC matrix reasoning scales were completed, those IQ scales are reported here. In addition, the British Picture Vocabulary Scale (BPVS3; Dunn and Dunn, 2009) was administered to assess receptive vocabulary and the Phonological Assessment Battery (PhAB, Frederickson et al., 1997; GL Assessment) was administered to assess phonological awareness at the rhyme and phoneme levels, along with rapid naming of objects and digits (RAN). The children’s rise time thresholds were measured using 3 psychoacoustic threshold tasks, one based on sine tones, one based on tones made from speech-shaped noise, and one using a synthetic syllable “ba”.

1. *British Ability Scales* (BAS)*, Reading and Spelling Tests*. The BAS is a set of standardized cognitive tasks that includes measures of single word reading and spelling. Children are presented with a sheet of single words that increase in difficulty to read aloud or are dictated a set of single words to spell that increase in difficulty, both with no time pressure. Assessment is halted after 6 consecutive errors, and standard scores can be computed with a mean of 100, S.D. 15.
2. *Test of Word Reading Efficiency* (TOWRE). The TOWRE consists of two subtests measuring speeded decoding of words (SWE, Sight Word Efficiency) and nonwords (PDE, Phonetic Decoding Efficiency). In each case, children are required to read aloud from a list of items graded in difficulty as many items as they can manage in 45 s, as quickly and as accurately as possible. Standard scores can be computed with a mean of 100, S.D. 15.
3. *British Picture Vocabulary Scales* (BPVS, Dunn and Dunn, 2009). The child is shown 4 pictures per trial provided in a stimulus book, and the administrator says a word. The child must point to the picture that best illustrates the meaning of that word. A standard score can be computed with a mean of 100, S.D. 15.
4. *Wechsler Intelligence Scales for Children* (WISC, Wechsler, 2016). Four of the core sub-tests of the WISC IV were used as measures of verbal versus non-verbal reasoning respectively, since a full-scale IQ can be estimated on this basis (Aubry & Bourdin, 2018). Two measures were completed at the time of EEG data collection. One was the verbal *Similarities* sub-test, where the child is given two words orally that name common objects or concepts and is asked to say how they are similar. The other was the non-verbal *Matrix Reasoning* sub-test, where the child is presented with a matrix of abstract pictures in which there is one picture missing. She/he has then to choose the missing picture from a number of possible options. Note that given their phonological difficulties, children with dyslexia may differ from typically-developing control children for verbal IQ measures, but they should not differ from controls for non-verbal estimates of IQ.
5. *Phonological Assessment Battery* (PhAB) *Rhyme and Phoneme Sub-scales*. The *Rhyme* Test was used to assess the children’s ability to identify the rime in single syllable words. Participants listened to an administrator say three words and then chose which two of the three words ended with the same shared sound (e.g. *big*, *hiss, miss*). The *Spoonerisms* Test was used to make an assessment of the children’s ability to isolate phonemes in single syllable words and then recombine them to make new words. In Part 1 of the test the children were asked to replace the first sound of a word with a new sound, in order to make a new word. For example, ‘cat’ with a ‘f’ makes ‘fat’. In Part 2 of the test the children were asked to exchange the first sounds of two words to make two new words. For example, ‘lazy dog’ makes ‘daisy log’. For each task, a standard score can be computed with a mean of 100 and S.D. of 15.

### 2.3 Psychoacoustic threshold rise time tasks

By Temporal Sampling theory, atypical neural entrainment in dyslexia is related to impaired auditory sensitivity to amplitude rise times. Three tasks were created to assess children’s sensitivity to rise times, based on a cartoon interface AXB task described previously by Goswami et al. (2021). In brief, the tasks were presented as child-friendly computer games based on the 3I-2AFC “Dinosaur” threshold estimation program originally developed by Dorothy Bishop (Oxford University). The threshold estimation program used an adaptive staircase procedure (Levitt, 1971) with an initial 2-down 1-up procedure followed by 3-down 1-up procedure after two reversals. The initial step size was eight, which halved after the fourth and sixth reversal. A run of the program terminated after the eighth reversal or 40 trials, whichever occurred first. The threshold score was calculated using the mean of the last 4 reversals. The stimuli were based on (1) simple sine tones (see Goswami et al., 2021); (2) unmodulated speech-shaped noise (SSN) with the long-term average spectrum of speech (CCITT Rec. 227), created from Gaussian noise by the penultimate author using MATLAB ccitt_filter function (https://www.auditory.org/mhonarc/2005/msg00098.html), and (3) the synthesised syllable /ba/ which was 300 ms long with a flat f0 (fundamental frequency) of 200 Hz (see Cumming et al., 2015).

### 2.4 Auditory-visual task and rhythmic delivery

**Fig. 1:**
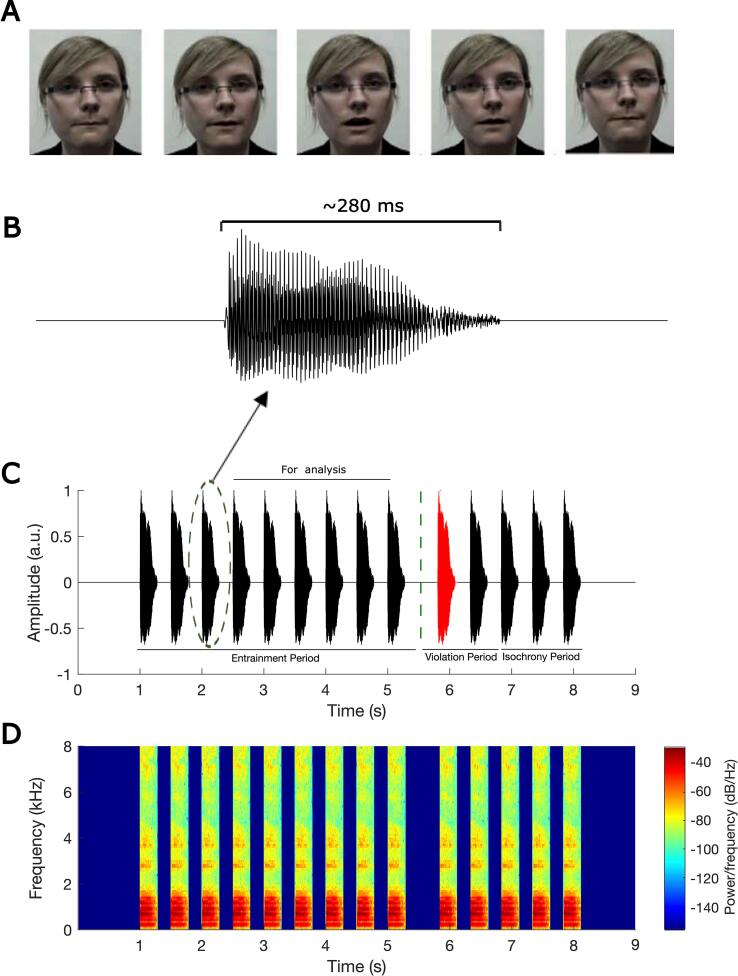
Experimental Design: (A) frames of the visual stimulus corresponding with a syllable “ba”, (B) waveform of a single auditory “ba”, (C) a rhythmic sequence of “ba” with the oddball as stimulus 10, and (D) the spectrogram corresponding to the rhythmic sequence of “ba”. The auditory stimuli consisted of the syllable “ba” repeated 14 times per trial at a rate of 2 Hz, with one syllable of stimuli 9–11 being out of the rhythm (here syllable 10, depicted in red). For this example, data analysis focused on stimuli 4–9 of the entrainment period.

The stimuli and experimental set-up were identical to the auditory-visual (AV) condition of the rhythmic entrainment task described by Power et al., 2012, Power et al., 2013, and following that study we analysed phase consistency using all EEG channels and using Cz as the reference channel. The children listened to a rhythmic sequence of the syllable “ba” that was repeated 14 times per trial, at a repetition rate of 2 Hz. Rhythmic violation depended on delaying the occurrence of a “ba” in the isochronous stream. One of the 14 syllables (at either position 9, 10 or 11) could be out of time in each sequence, in an unpredictable randomised order. Each participant was presented 90 trials, 15 of which did not contain a violation and were presented randomly as catch trials. Each trial consisted of three periods: the entrainment period (stimuli 1 – 8 or 1 – 9 depending on oddball location; see Fig. 1), the violation period (one of stimuli 9 – 11 violated the isochronous rhythm) and the return-to-isochrony period (stimuli 12 – 14). The “ba” stimuli in the entrainment period were presented with a fixed time interval of 500 ms. The degree to which the violator was out of the isochronous rhythm varied depending on how well the child was doing in the task. If a child correctly identified three violators in three consecutive sequences, then the deviation from 500 ms stimulus-onset asynchrony was reduced by 16.67 ms. If a violator was not detected, then the deviation increased by 16.67 ms. This 3-down 1-up staircase procedure was employed to equate task engagement and performance across participants. Theoretically the 3-down 1-up staircase should result in a performance accuracy of 79.4% for each participant (Levitt, 1971). The time interval between successive trials was 3 s. For the current analyses, we considered the entrainment period only. To ensure that rhythmicity had been satisfied and to maximise data, we excluded the first two “ba” stimuli for sequences with a violation occurring at position 9 (hence analyses utilised six stimuli, 3 – 8) and the first three “ba” stimuli for sequences where the violation occurred at position either 10 or 11 (hence analyses utilised six stimuli, 4 – 9). This yielded 6 entrainment stimuli for each of the 90 trials rather than the 5 stimuli used by Power et al., 2012, Power et al., 2013, resulting in a maximum of 540 stimuli.

### 2.5 EEG data acquisition

Participants were seated in a soundproof room. The auditory stimuli (through earphones at both ears) and visual speech information (video of a “talking head”) were presented to the participant while EEG data were collected at a sampling rate of 1 kHz using a 128-channel EEG system (HydroCel Geodesic Sensor Net). Visual cues started 68 ms before the onset of the stimulus “ba” as in natural speech. Participants were instructed to concentrate their gaze on the lips of the female face and to listen to the auditory stimuli. They were asked to press a target key on the keyboard if one of the syllables was out of time, breaking the rhythm. The total recording time for the EEG study (excluding the set-up) was about 15 min.

### 2.6 EEG data pre-processing

The collected EEG data were referenced to Cz channel and then band-passed filtered into frequency range of 0.5 – 48 Hz using a zero phase FIR filter with low cutoff (−6 ​dB) of 0.25 ​Hz and high cutoff (−6 dB) of 48.25 Hz (EEGLab Toolbox; Delorme and Makeig, 2004). Bad channels were detected and interpolated through spherical interpolation (EEGLab Toolbox). In particular, channels with extreme noise were detected using the spectrogram, kurtosis and probability methods provided by EEGLAB Toolbox. A channel was labelled for rejection if it was 3 S.D. away from the average and was then interpolated using spherical interpolation (Delorme and Makeig, 2004). We ran the independent component analysis, provided in EEGLab Toolbox, on the data for each participant. The calculated independent components were then assessed carefully to remove artefactual components such as eye blinks and eye movements. We also monitored participant head movements during the EEG recording through a camera installed in the EEG room and the times corresponding to head movements were recorded by the experimenter. This information was then used to ensure that trials corrupted by head movements had been cleaned by the pre-processing steps. The EEG data were downsampled to 100 Hz and band-pass filtered to extract delta (0.5 – 4 Hz), theta (4 – 8 Hz) and alpha (8 – 12 Hz) frequency bands (MNE Library-Python, Gramfort et al., 2013). The data were then epoched (windowed) into individual trials, in the time range of 500 ms before the onset of the first stimulus and 4.5 sec after that. Because of technical issues (such as stimulus information not marked in the EEG file), some trials were excluded. The average number of trials used for the analyses for control and dyslexic groups were 77.61 (S.D. = 20.01) and 78.13 (S.D. = 21.67), respectively. We next determined the instantaneous phase separately for each EEG channel and for each frequency band using two steps: (1) Calculating the analytic representation (complex form) of the input signal through the Hilbert transform; (2) Calculating the phase of each sample (which is a complex value) of the analytic signal. Note that the focus of the reported analyses was only on the entrainment period (3rd-8th or 4th-9th stimuli). We did not apply any analyses to the violation and isochrony periods.

### 2.7 Computation of phase entrainment

To assess the phase entrainment for each group in each frequency band (delta, theta, alpha), we performed the following steps: (1) Calculating instantaneous phases of all 128 EEG channels at the times corresponding to the onsets of the 6 “ba” stimuli that were used for the analyses (e.g. stimuli 4 – 9 for the sequence shown in Fig. 1) for each of the 90 trials; (2) Calculating the mean phase for each of the 90 trials by averaging across the phase observations obtained for 128 EEG channels and for the 6 “ba” stimuli in step 1; This results in a single phase value (for example, 1.5 rad) for each trial. (Note that the mean of a set of phase values cannot be calculated by arithmetic averaging across the values (Berens, 2009). For example, considering three phase values (in degree): 25°, 15° and 350°, the arithmetic mean of these values is equal to 130° while all phase samples point towards 0°). (3) Deriving a single unit vector (whose angle is determined by the phase value obtained in step 2) in the vector space for each trial; (4) Calculating the mean vector for each child by averaging across the unit vectors obtained in step 3; This results in a single vector for each child, subsequently we refer to this as the *child resultant vector*. The length of the *child resultant vector* is a value between 0 and 1 and its angle, called *child preferred phase*, is between 0 and 2 π. The length of the *child resultant vector* can be used as a criterion to assess the strength of phase consistency across different trials for each individual participant. As a next step (5), a single unit vector (whose angle is determined by the *child preferred phase* obtained in step 4) is considered in the vector space for each child; and then (6) the mean vector for each group is computed by averaging across the unit vectors obtained in step 5. This produces a single vector, called the *group resultant vector*, for each group. The length of the *group resultant vector* is a value between 0 and 1 and its angle, called *group preferred phase*, is between 0 and 2 π. The length of the *group resultant vector* can be used as a criterion to assess the strength of phase consistency across different children by group.

### 2.8 Statistical analysis

To investigate the entrainment phase consistency, the Rayleigh test of uniformity was applied to the *child preferred phases* separately for frequency bands of interest and for each group. The Watson's *U*^2^ test was also used to see if the phase distribution fitted well with the von Mises distribution. To compare the strength of phase entrainment consistency in the control group with the dyslexic group, a Wilcoxon rank sum test was used, conducting separate tests based on the length of the *child resultant vectors* for the frequency band of interest. To compare the preferred phases of the control group with the dyslexic group at the onset of “ba” stimuli, the one sample test for the mean angle was applied for frequency bands of interest. We further investigated the behaviour of phase and phase changes across time (angular velocity), prior to and just after the occurrence of a stimulus over the time interval of –500 ms to 500 ms, for each group and for each frequency band of interest. We then applied a two-sample *t*-test to assess the statistical difference in terms of per-stimulus angular velocity in the frequency band of interest. To explore the neural preparation for the next event in the stimulus stream for each group and each band, we calculated the ERP and then applied a two-sample *t*-test to assess the statistical difference between the two groups for the frequency band of interest. Finally, we explored relations between the *child preferred phase* and the length of the *child resultant vectors* in the frequency bands of interest and measures of reading and phonology using circular-linear and Pearson correlations, respectively. Multiple corrections were applied as appropriate. For the analyses designed to test specific hypotheses, no corrections for multiple comparisons were applied.

## 3 Results

### 3.1 Phase entrainment consistency within each group (dyslexic, control)

**Fig. 2:**
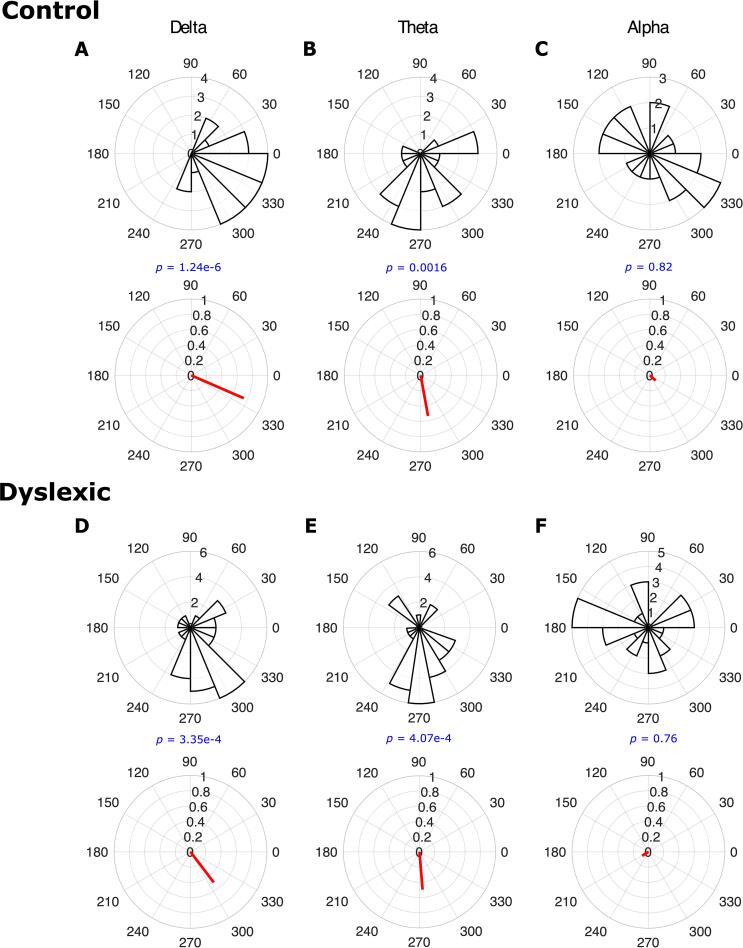
Phase distribution across groups (rose plots, number of bins = 15) and group resultant vectors (red lines). There is significant phase entrainment across different children in the control group for the delta (panel A) and theta (panel B) bands, but not for the alpha band (panel C). Consistent phase entrainment was also found in the dyslexic group (N = 30) for the delta (panel D) and theta (panel E) bands, but not for the alpha band (panel F).

To assess whether phase entrainment was consistently present across the different children in each group and in each frequency band, we applied the Rayleigh test of uniformity to the *child preferred phases* (angles of *child resultant vectors*, see step 4 in subsection 2.7) separately for each frequency band and for each group. For the control group, the Rayleigh test revealed that the distribution of phase at the onsets of the “ba” stimuli was significantly different from the uniform distribution for the delta band (Rayleigh test, *z* = 11.72, *p* = 1.24 × 10^-6^; see Fig. 2A) and also the theta band (Rayleigh test, *z* = 6.09, *p* = 0.0016; see Fig. 2B). Accordingly, phase entrainment for children in the control group was consistent in the delta and theta bands. The Watson's *U*^2^ test (Watson, 1961) also showed that the phase distribution could be fitted well with a unimodal von Mises distribution (a von Mises distribution is equivalent to a normal distribution in the circular/angular domain, see Mardia and Jupp, 2000); for the delta band (Watson's *U*^2^ test, *U*^2^ = 0.068, *p* = 0.52) and for the theta band (Watson's *U*^2^ test, *U*^2^ = 0.10, *p* = 0.25). For the alpha frequency band, however, we found no consistent phase entrainment (Rayleigh test, *z* = 0.21, *p* = 0.82; see Fig. 2C), accordingly we did not perform the Watson’s test. The children with dyslexia also showed consistent phase entrainment for the delta band (Rayleigh test, *z* = 7.6, *p* = 3.35 × 10^-4^; see Fig. 2D) and for the theta band (Rayleigh test, *z* = 7.43, *p* = 4.07 × 10^-4^; see Fig. 2E). This was not the case for the alpha band (Rayleigh test, *z* = 0.28, *p* = 0.76; see Fig. 2F). We again found that the phase distribution could be fitted well with a von Mises distribution for the delta band (Watson's *U*^2^ test, *U*^2^ = 0.09, *p* = 0.32) and for the theta band (Watson's *U*^2^ test, *U*^2^ = 0.08, *p* = 0.40). Accordingly, both groups of children showed consistent phase entrainment in both lower-frequency bands.

As noted, the arrival of Covid-19 prevented the original experimental design of this study being fulfilled, in which group sizes were equal. In order to ensure that our findings were robust against the different number of participants in each group, we also selected the 21 dyslexic children with the lowest scores on the behavioural (phonological and reading) tasks. The same phase consistency analyses were employed. Similar results were found, with consistent phase entrainment for the dyslexic group for the delta band (Rayleigh test, *z* = 5.49, *p* = 0.0032) and for the theta band (Rayleigh test, *z* = 5.55, *p* = 0.003), but not for the alpha band (Rayleigh test, *z* = 0.04, *p* = 0.96). Accordingly, children in both the dyslexic and control groups showed evidence of consistent phase entrainment in the delta and theta bands. Figures matching those presented here for these N = 21 comparisons are presented in the Supplementary Materials (please see Fig. S1, which matches Fig. 2).

### 3.2 Comparing the strength of the consistency of phase entrainment between groups

**Fig. 3:**
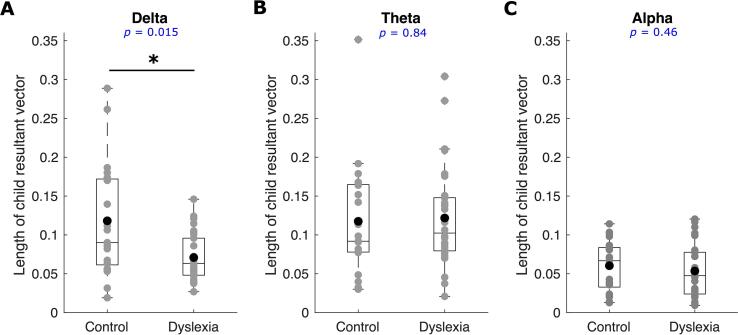
The length of child resultant vectors was used to compare the strength of phase consistency in the control group with the dyslexic (N = 30) group. The grey disks denote the length of *child resultant vector* for individual children, the black disks denote the mean values, and the horizontal line on each box indicates the median. The phase entrainment in the delta band (panel A) for the control group was significantly greater than that of the dyslexic group, while no significant group differences were found for the theta band (panel B) nor for the alpha band (panel C).

The length of the *child resultant vector* (step 4 in subsection 2.7) for a given child can be considered as an indicator of phase entrainment consistency across different trials for that participant, with longer *child resultant vectors* indicating higher consistency of phase entrainment. Comparison of Fig. 2A and 2D suggests different resultant vector lengths by group. To compare the consistency of phase entrainment in the control group with the dyslexic group, we used a Wilcoxon rank sum test, conducting separate tests based on the length of the *child resultant vectors* for each frequency band (shown in Fig. 3). Phase entrainment in the delta band was significantly greater for the control group compared to the dyslexic group (Wilcoxon rank sum test, *z* = 2.4, *p* = 0.015; see Fig. 3A). However, there was no significant difference in phase entrainment between the two groups in the theta band (Wilcoxon rank sum test, *z* = – 0.2, *p* = 0.84; see Fig. 3B) nor in the alpha band (Wilcoxon rank sum test, *z* = 0.74, *p* = 0.46; see Fig. 3C). Accordingly, the children with dyslexia showed differential neural responding (significantly reduced phase consistency) in the delta band.

We repeated the Wilcoxon rank sum tests to compare the 21 children in the control group with the 21 dyslexic children with the lowest reading and phonology scores. Again, phase entrainment in the delta band was significantly greater for the control group compared to this subset of the dyslexic group (Wilcoxon rank sum test, *z* = 2.5, *p* = 0.012). Again, there was no significant difference in phase entrainment between the two groups for the theta band (Wilcoxon rank sum test, *z* = 0.17, *p* = 0.86) nor for the alpha band (Wilcoxon rank sum test, *z* = 1.2, *p* = 0.2; see Fig. S2).

### 3.3 Comparing preferred phase between groups

As will be recalled, *a priori* we had predicted a difference in *group preferred phase* (angle of *group resultant vector*, see step 6 in subsection 2.7) on the basis of Power et al. (2013). We used a 1-tailed test to compare the preferred phases of the control group with the dyslexic group. Visual inspection of the vector angles shown in Fig. 2 suggests a difference by group for the delta band only. We computed the one sample test for the mean angle, conducting separate tests for delta and theta bands (we excluded the alpha band as there was no consistent phase entrainment for this band in either group). The results showed that the *group preferred phase* of entrainment in the delta band (one sample test for the mean angle, *p* = 0.007) was significantly different. This was not the case for the theta band (one sample test for the mean angle, *p* = 0.14). Accordingly, neural responding in dyslexia in the delta band is aligned to a different temporal point in the input despite the predictable nature of the rhythmic speech stimuli. This was the same for the matched groups of N = 21, please see Fig. S1.

**Fig. 4:**
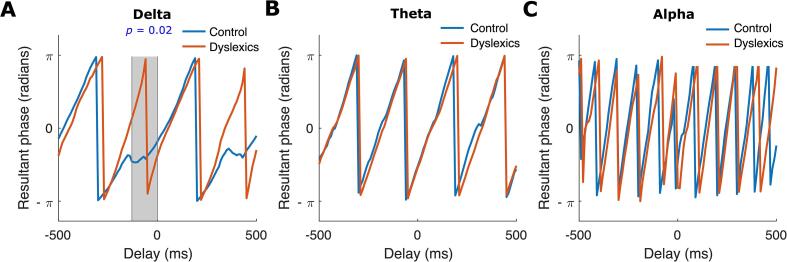
The group preferred phase (radians) versus the delay (ms) relative to “ba” stimuli onsets. The blue and red curves are for control and dyslexic (N = 30) groups, respectively. The curves related to the delta band (panel A) reveal a pre-stimulus difference between the control and dyslexic groups. In particular, the pre-stimulus angular velocity in the delta band over the time interval of –130 ms to 0 ms (marked with grey colour) differs significantly by group. Moreover, the control curve appears to follow a quasiperiodic pattern with a frequency of around 2 Hz while the dyslexic curve appears to follow a quasiperiodic pattern with a frequency rate of around 4 Hz. Such group differences are not observed in the theta band (panel B) nor in the alpha band (panel C), which also plot pre-stimulus *group preferred phase* in each case.

We then explored the behaviour of preferred phase regarding its changes across time in response to rhythmic (periodic) auditory stimuli separately for each group and each frequency band. Note that phase changes across time refers to angular velocity. We therefore calculated the *group preferred phase* (see step 6 in subsection 2.7) just prior to and just after the occurrence of a stimulus (stimuli 3–8 or 4–9) in the entrainment period over the time interval of –500 ms to 500 ms, separately for each frequency band and each group (control, dyslexic; see Fig. 4). Note that this time interval (1 s) corresponds to two repetitions of the syllable “ba”. Fig. 4A shows plots depicting the *group preferred phase* obtained for the two groups for the delta band. There is a clear visual difference between groups. The plot for the control group appears to follow a quasiperiodic pattern with a frequency of around 2 Hz (angular velocity of 4π rad/s). However, the plot for the dyslexic group appears to follow a quasiperiodic pattern with frequency rate of around 4 Hz (angular velocity of 8π rad/s). Hence for the children with dyslexia, the group angular velocity in the delta band appears to be almost twice the rate of the control group. In particular, visual inspection of the group data shown in Fig. 4A suggests that the pre-stimulus angular velocity in the delta band for the control group is notably slower than that of the dyslexic group (Fig. 4A, blue lines). We employed a two-sample *t*-test to find the time interval showing a statistical difference in terms of angular velocity. We found a significant difference between the two groups (two-sample *t*-test, *p* = 0.02; see Fig. 4A) over the time interval of –130 ms to 0 ms. Meanwhile, the curves for the two groups regarding angular velocity for the theta band and for the alpha band look very similar (Fig. 4B, 4C), and we did not find any time interval in which the two groups differed statistically, as predicted. Matched analyses for the smaller groups (N = 21) are presented in the Supplementary Materials and were similar (please see Fig. S3).

### 3.4 Event-related potentials

**Fig. 5:**
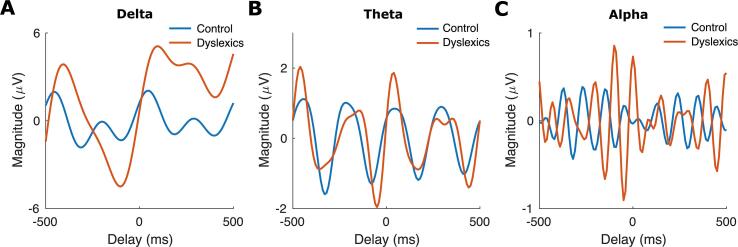
Event-related potentials. The blue and red curves denote the ERPs for control and dyslexic groups, respectively. Please note the difference in Microvolt (μV) on the Y axes for each plot. A clear difference can be observed between the ERP curve of the control group and that of dyslexic group for the delta band (panel A). In particular, the ERP curve for the control group follows a quasiperiodic pattern with a frequency of around 2 Hz. No group difference can be observed in the theta band (panel B) nor in the alpha band (panel C).

To further analyse the cognitive processes associated with the task, we calculated the ERPs in the time domain separately for each group and each frequency band in the time interval of –500 ms to 500 ms. The ERPs were computed by averaging across the voltage amplitudes (in Microvolt [μV]) of all 128 pre-processed EEG channels for all trials and all participants in each group. Different pre-stimulus ERPs would suggest differential preparatory brain activity between groups to the upcoming event in the rhythmic stimulus stream, even though presentation of the next “ba” stimulus in the entrainment period was rhythmically isochronous and therefore entirely predictable. Soltész et al. (2013) found significantly less neural preparation over the time interval of –40 ms to 0 ms for the next event in the tone stimulus stream for their adult dyslexic participants. To examine this parameter in children, we also checked pre-stimulus neural preparation. However, we did not find a significant difference between our child groups regarding the pre-stimulus activity over the interval of –40 ms to 0 ms, related to expectancy of the next periodic stimulus (Outlier removed, two-sample *t*-test, *p* = 0.34). As a further test, we also calculated the delta band ERP for stimuli 1–2, as differences in ERP amplitude due to expectancy are often strongest for the first two stimuli of a periodic input (Fig. S4). We then applied a two-sample *t*-test to compare the neural preparation between the two groups. We did not a significant difference (outlier removed, two-sample *t*-test, *p* = 0.46). The ERP data are shown in Fig. 5 (see Fig. S5 for the N = 21 matched groups). A clear difference can be observed between the ERP curves obtained for the control and dyslexic participants for the delta band (Panel A). The curve for the control group has a quasiperiodic pattern with a frequency of around 2 Hz; the curve for the dyslexic group does not. The broadband (0.5 – 48 Hz) ERP for each group is also shown in Fig. S6.

### 3.5 Brain-behaviour correlations

Finally, we explored relations between *child preferred phase* and the length of the *child resultant vectors* (in the delta and theta bands) and the behavioural measures of reading, language, auditory processing and phonology. To achieve this, we first created scatter plots for these variables (and for age and IQ) for both EEG measures (shown in Figs. S7–S10, Supplementary Materials). As circular-linear correlations are required for the phase measures, whereas Pearson correlations are appropriate for the child resultant vector length measures, we were only able to compute slopes for each group for the resultant vector length plots (see Figs. S9 and S10). For these scatterplots, we next applied a linear regression model for each *pair* of child resultant vector length-behavioural measures to fit a linear line. Student’s t-tests were then used to explore whether the slope of fitted line for the control group was significantly different from that of the dyslexic group for each pair (the *p*-values are provided in Table S1). If the slopes for a given pair were not statistically different, we then computed the Pearson correlation across all children for that pair of measures (see Table 2). The only significant associations found were between the length of the child resultant vector in the delta band and (a) timed single word reading (TOWRE SWE, *r* = 0.32, *p* < 0.05), (b) timed nonword reading (TOWRE PDE, *r* = 0.41, *p* < 0.01), and (c) RAN (digits, *r* = 0.30, *p* < 0.05). In each case, children with longer resultant vectors showed better behavioural performance. We also ran correlations for the children with dyslexia and the control children separately, these are shown in Table S2.

**Table 2:** Pearson correlations between child resultant vector length and the behavioural measures of reading and phonology that showed slopes by group that were not statistically different (see Figs. S9 and S10) (N = 51).

	Age (months)	WISC Sim	WISC Matrix	BPVS	BAS Reading SS	BAS Reading Age	BAS Spelling SS	TOWRE SWE SS	Nonword Reading SS	PhAB Rhyme SS	PhAB Phoneme	RAN Pictures	RAN Digits	Rise time Sine	Rise time SSN	Rise time “ba”
Delta - length of *child resultant vector*	–	–0.03	–0.06	–	–	–	0.25	0.32*	0.41**	–	0.25	0.21	0.30*	– 0.04	–0.01	–
Theta - length of *child resultant vector*	0.21	–0.07	–0.15	–	–	–	–	0.04	0.14	–0.12	0.25	0.04	0.08	– 0.02	–0.07	– 0.02

Note. **p < 0.01; *p < 0.05. BAS Reading SS = British Ability Scales standardized score; Reading Age = from BAS; BAS Spelling SS = British Ability Scales standardized score; TOWRE SWE SS = TOWRE Single Word Efficiency standardized score; TOWRE Nonword reading SS = TOWRE Phonic Decoding Efficiency Scale standardized score = 100; PhAB Rhyme = Phonological Awareness Battery Rhyming standardized score; PhAB Phoneme = Phonological Awareness Battery Spoonerism standardized score; RAN pictures = Phonological Awareness Battery Picture Naming standardized score; RAN digits = Phonological Awareness Battery Digit Naming standardized score; Rise time SSN = threshold in rise time task based on the speech-shaped noise stimuli in ms; Rise time sine = threshold in rise time task based on sine tone stimuli in ms, Rise time “ba” = threshold in rise time task based on the syllable “ba” in ms.

Regarding the correlations for preferred phase, more significant relations were found for the control group than for the dyslexic group (see Table S2). Control children showed significant correlations between delta preferred phase and RAN (picture naming, *r* = 0.55, *p* < 0.05), and between theta preferred phase and single word reading (*r* = 0.54, *p* < 0.05), rhyme awareness (*r* = 0.57, *p* < 0.05), and rise time sensitivity (for SSN, *r* = 0.68, *p* < 0.01). Children with dyslexia also showed a significant correlation between theta preferred phase and rhyme awareness (*r* = 0.50, *p* < 0.05). However, none of these correlations survived correction for multiple comparisons.

## 4 Discussion

The data reported here provide the first steps to understanding which neural mechanisms may contribute to the atypical speech-brain alignment that has been reported in speech processing studies that include children with developmental dyslexia (Power et al., 2013, Power et al., 2016, Molinaro et al., 2016, Di Liberto et al., 2018, Destoky et al., 2020). Adopting the simple rhythmic repetitive speech paradigm developed by Power et al., 2012, Power et al., 2013, the analyses of delta band phase entrainment utilised here revealed that the dyslexic child brain showed atypical responding compared to the typically-developing child brain. The previous findings reported by Power et al. (2013) regarding group differences in preferred phase in the delta band were replicated, and we showed with novel analyses that phase changes across time as measured by angular velocity also differed between groups. The dyslexic brains failed to exhibit the same pre-stimulus (time interval of –130 ms to 0 ms, Fig. 4A) angular velocity as the typically-developing brains. Regarding the strength of phase entrainment by group, the lengths of the child resultant vectors were significantly different between groups for the delta band only (Fig. 3A), consistent with impaired phase entrainment in dyslexia in the delta band. This is also novel, as Power et al. (2013) did not find a group difference in strength of phase consistency. This difference may be due to the difference in age of the participants in the two studies (13-year-olds in Power et al., 2013, 9-years-olds here). The finding that both 9-year-old children with dyslexia and 13-year-old children with dyslexia (Power et al., 2013) show a different preferred phase of entrainment in the delta band compared to age-matched typically-developing control children is suggestive of less efficient neural processing of rhythmic speech in dyslexia. Neural responding in the delta band was aligned to a different temporal point for each group, despite the highly predictable rhythmic input (visible in Fig. 2A versus 2D). This suggests that, at least for relatively slow rhythmic speech information (syllables delivered at a 2 Hz rate), accurate and efficient stimulus processing is impeded for children with developmental dyslexia because their brains utilise a different preferred phase of neural entrainment.

Further analyses of the group preferred phase data (Fig. 4) were suggestive of a quasi-periodic pattern in the resultant phase analyses. While the group of typically-developing children had an average frequency of around 2 Hz, consistent with the input, the quasi-periodic pattern for the dyslexic group in the resultant phase analyses had a frequency of around 4 Hz, twice the rate of the input. Meanwhile, the ERP analyses (Fig. 5A) suggested that the dyslexic brain may be impaired in the *temporal prediction* of speech information in the delta band, at least for speech delivered at an isochronous rate of 2 Hz. In particular, the ERP curve for the control group followed a quasi-periodic pattern with a frequency (almost 2 Hz) that was similar to the stimuli, while the curve for the dyslexic group did not. However, the pre-stimulus activity over the interval of –40 ms to 0 ms did not show a significant difference between the two groups, in contrast to prior data with adult dyslexics. Soltész et al. (2013) reported significantly less neural preparation for the next event in a tone stimulus stream for their adult dyslexic participants, but our data did not replicate this reduced preparation with children. This could reflect factors such as the generally noisier EEG data that is typical of child participants, a genuine difference regarding temporal prediction between children and adults with dyslexia, or our use of speech stimuli rather than the tone stimuli used by Soltész et al. (2013).

The differences in phase entrainment in the delta band revealed here are likely to have serious consequences for linguistic processing by children with developmental dyslexia. Given that temporal prediction during speech processing across languages may involve temporal markers every 2 Hz (the rate of stressed syllable production across languages, see Kotz et al., 2009, Dauer, 1983), and given that infant-directed speech is known to enhance the acoustic salience of these delta-band temporal markers (Leong et al., 2017), impairment of phase entrainment and temporal prediction in the delta band is likely to impair the development of speech processing by infants at genetic risk of dyslexia from birth. Typically-developing infants learning both English and German are known to show delta-band entrainment early in life (Attaheri et al., 2022, Telkemeyer et al., 2011). Any mechanistic impediment to efficient cortical entrainment to the speech signal in the delta band is thus likely to have consequences for both speech processing and the development of an efficient phonological system (Goswami, 2019, for a recent review). As noted earlier, by Temporal Sampling theory atypical encoding of low-frequency information in the speech signal relevant to extracting speech rhythm and prosody is proposed to underlie the ‘phonological deficit’ found in dyslexia across languages (Goswami, 2011). As prosodic structure is a feature of all languages, whether their rhythm is stress-timed, syllable-timed or has moraic timing, the impaired neural mechanisms identified here could contribute to explaining the aetiology of impaired phonological processing in developmental dyslexia in all languages and orthographies.

Further research using simple rhythmic repetitive speech paradigms in other languages could throw light on whether the impaired mechanisms of temporal prediction and phase alignment revealed here using English characterise children with developmental dyslexia in other languages. The simple nature of the task also opens the way to neural studies of infants at genetic risk for developmental dyslexia (see Kalashnikova et al., 2018). The rhythmic speech task has already been validated as a measure of individual differences in EEG studies with typically-developing infants (Gibbon et al., 2021), albeit using a passive listening paradigm where no overt responding by listening infants was required. Accordingly, a rhythmic repetitive speech task could enable a simple early neural marker of later dyslexia. Similar neural paradigms may also prove useful for investigating the mechanistic basis of other developmental disorders of language, such as DLD and stuttering. Atypical rhythm processing has been hypothesised to play a key role in these other disorders (Ladányi et al., 2020), and has been shown to be impaired in behavioural studies of children with DLD (Cumming et al., 2015). Identifying the atypical neural mechanisms that contribute to developmental disorders of language would also enable the development of novel remediation programmes, for example involving rhythm (Goswami and Szűcs, 2011, Bhide et al., 2013, Fiveash et al., 2021).

In conclusion, we show here for the first time that the dyslexic brain is characterised by significantly reduced phase consistency and atypical pre-stimulus angular velocity when processing incoming isochronous and predictable rhythmically-produced speech stimuli. Neural responding to simple beat-based stimuli may thus offer a simple diagnostic tool for identifying developmental dyslexia, which could even be used to assess dyslexia risk status during infancy (Gibbon et al., 2021). Such a task would also be language-neutral, enabling its use across languages with different linguistic rhythm types. Given that the neural response being measured is automatic and an inherent aspect of sensory processing, such a diagnostic measure would also be resistant to differences in children’s attentional and cognitive capacities.

## CRediT authorship contribution statement

**Mahmoud Keshavarzi:** Conceptualization, Methodology, Data analysis, Visualization, Writing – original draft. **Kanad Mandke:** EEG Methodology, Investigation, Writing – review & editing. **Annabel Macfarlane:** Investigation, Data curation. **Lyla Parvez:** Investigation. **Fiona Gabrielczyk:** Investigation, Data curation. **Angela Wilson:** Investigation, Data curation. **Usha Goswami:** Funding acquisition, Conceptualization, Methodology, Resources, Supervision, Writing – original draft.

## Declaration of Competing Interest

The authors declare that they have no known competing financial interests or personal relationships that could have appeared to influence the work reported in this paper.
