# Supplementary material for: Atypical delta-band phase consistency and atypical preferred phase in children with dyslexia during neural entrainment to rhythmic audio-visual speech
Source: Neuroimage Clin. 2022 May 20;35:103054. doi: 10.1016/j.nicl.2022.103054 (PMC9136320; doi:10.1016/j.nicl.2022.103054)
Supplement: Supplementary data 1 [file mmc1.docx]

**Supplementary Information**

**Figure S1. As Figure 2 but for 21 children with dyslexia who scored lowest on the phonological and behavioural measures.**

**Figure S2**. **As Figure 3 but for 21 children with dyslexia who scored lowest on the phonological and behavioural measures.**

**Figure S3**. **As Figure 4 but for 21 children with dyslexia who scored lowest on the phonological and behavioural measures**.

**Figure S4.** **Delta-band time-domain event-related potential for stimuli 1st-2nd.** The blue and red curves denote the ERPs for control and dyslexic groups, respectively.

**Figure S5**. **As Figure 5 but for 21 children with dyslexia who scored lowest on the phonological and behavioural measures.**

**Figure S6. Broad-band time-domain event-related potentials.** The blue and red curves denote the ERPs for control and dyslexic groups, respectively.

**Figure S7.** **Scatter plot of the *child preferred phase* in the delta band and behavioural measures.**

**Figure S8.** **Scatter plot of the *child preferred phase* in the theta band and behavioural measures.**

**Figure S9. Scatter plot of length of *child resultant vectors* in the delta band and behavioural measures.**

**Figure S10. Scatter plot of length of *child resultant vectors* in the theta band and behavioural measures.**

Table S1. The p-values (Student’s t-tests) comparing the slopes obtained from the linear regression models for the child resultant vector comparisons between the two groups.

|  | Age  (months) | WISC Sim | WISC Matrix | BPVS | BAS  Reading SS | BAS  Reading Age | BAS  Spelling SS | TOWRE SWE SS | Nonword Reading SS | PhAB Rhyme SS | PhAB Phoneme | RAN Pictures | RAN Digits | Rise time Sine | Rise time SSN | Rise time “ba” |
| --- | --- | --- | --- | --- | --- | --- | --- | --- | --- | --- | --- | --- | --- | --- | --- | --- |
| Delta - length of *child resultant vector* | .00 | 1 | .28 | .00 | .00 | .00 | 1 | .07 | 1 | .00 | 0.28 | 1 | .72 | 1 | 1 | .00 |
| Theta - length of *child resultant vector* | .11 | 1 | 1 | .00 | 00 | .03 | .00 | .12 | .69 | 1 | .50 | 1 | .98 | .37 | .08 | .49 |

Note. Reading SS = British Ability Scales standardized score; Spelling SS = British Ability Scales standardized score; Nonword reading SS = TOWRE Phonic Decoding Efficiency Scale standardized score = 100; PhAB Phoneme = Phonological Awareness Battery Spoonerism standardized score; RAN pictures = Phonological Awareness Battery Picture Naming standardized score; Rise time “ba” = threshold in rise time task based on the syllable “ba” in ms; Rise time sine = threshold in rise time task based on sine tone stimuli in ms.

Table S2. Circular-Linear and Pearson correlations between the neural phase measures and the behavioural measures, showing variables with non-significantly different slopes only, by group (Dyslexic N = 30, Control N = 21).

|  | | Age  (months) | WISC Sim | WISC Matrix | BPVS | BAS  Reading SS | BAS  Reading Age | BAS  Spelling SS | TOWRE SWE SS | Nonword Reading SS | PhAB Rhyme SS | PhAB Phoneme | RAN Pictures | RAN Digits | Rise time Sine | Rise time SSN | Rise time “ba” |
| --- | --- | --- | --- | --- | --- | --- | --- | --- | --- | --- | --- | --- | --- | --- | --- | --- | --- |
| Delta - *child preferred phase* | C | .30 | .26 | .32 | .38 | .07 | .09 | .20 | .41 | .09 | .11 | .31 | .55* | .10 | .18 | .12 | .23 |
|  | D | _._29 | .17 | .21 | .24 | .20 | .22 | .32 | .15 | .24 | .21 | .22 | .28 | .42 | .29 | .25 | .20 |
| Theta - *child preferred phase* | C | .42 | .22 | .24 | .25 | .54* | .22 | .29 | .34 | .18 | .57* | .33 | .35 | .52^+^ | .35 | .68** | .33 |
|  | D | .36 | .42 | .35 | .19 | .28 | .25 | .37 | .04 | .05 | .50* | .15 | .38 | .22 | .20 | .22 | .22 |
| Delta - length of *child resultant vector* | C | - | -.34 | -.01 | - | - | - | -.17 | -.02 | .26 | - | .04 | .13 | .14 | .07 | .03 | - |
|  | D | - | -.04 | -.06 | - | - | - | -.12 | .14 | .2 | - | .27 | .08 | .26 | .02 | .00 | - |
| Theta - length of *child resultant vector* | C | -.10 | -.12 | -.27 | - | - | - | - | -.02 | .24 | -.26 | .26 | .10 | .15 | -.02 | .07 | -.03 |
|  | D | .44* | -.03 | -.06 | - | - | - | - | .16 | .23 | -.07 | .30 | .02 | .06 | -.04 | -.18 | -.04 |

Note. ***p* < .01; **p* < .05. C = Control; D = Dyslexic; Reading SS = British Ability Scales standardized score; Reading Age = from BAS; Spelling SS = British Ability Scales standardized score; TOWRE SWE SS = TOWRE Single Word Efficiency standardized score; TOWRE Nonword reading SS = TOWRE Phonic Decoding Efficiency Scale standardized score = 100; PhAB Rhyme = Phonological Awareness Battery Rhyming standardized score; PhAB Phoneme = Phonological Awareness Battery Spoonerism standardized score; RAN pictures = Phonological Awareness Battery Picture Naming standardized score; RAN digits = Phonological Awareness Battery Digit Naming standardized score; Rise time SSN = threshold in rise time task based on the speech-shaped noise stimuli in ms; Rise time sine = threshold in rise time task based on sine tone stimuli in ms, Rise time “ba” = threshold in rise time task based on the syllable “ba” in ms.
